# Southern Dental Association

**Published:** 1885-06

**Authors:** 


### THIRD DAY.

Thursday, April 2, being Dentist's Day at the World's Cotton Centennial Exposition, no regular sessions were held.

Assembling at Tulane Hall at 9 A. M., the visiting and resident dentists with their lady friends proceeded in a body, some two hundred and fifty strong, to the foot of Canal street, where a steamboat waited to convey them up the Mississippi river to the Exposition grounds. Their crescent shaped badges attracted much attention, and the exclamation "what fine looking men" was frequently heard. The trip was a delightful one, affording a fine view of the busy city, with its warehouses and elevators lining the river edge; its many tall steeples in the distance; the broad streets forming avenues of living green, converging to a common centre, like the sticks of an open fan, from the crescent shaped city front. On the opposite side of the river lie the suburban villages of Gretna and Algiers, while lining the banks on either side lie at anchor vessels from across the broad ocean, and steamers from a hundred tributary streams. Reaching the Exposition landing, a halt was called on the margin of a little lake, under the shade of a gigantic live oak tree, draped with waving streamers of gray moss, where the group was photographed, facing the Exposition buildings, with the Mexican headqnarters in the rear. Thence they repaired to the Music Hall, in the main building, where front seats had been reserved for their use. Seated on the platform, and grouped around the speaker's stand, were representatives of the Board of Management of the Exposition and a number of the most prominent members of the dental profession. An address of welcome on the part of the dentists of New Orleans was delivered by Dr. A. G. Fredricks, and responded to by Dr. A. O. Rawls of Kentucky. An eloquent and appropriate address of welcome and congratulation, from Capt. S. H. Buck, Acting Director-General of the Exposition, was echoed by Col. F. C. Morehead, Commissioner-General. After brief remarks from others, and music, they repaired to the banks of Lake Rubio to witness a special drill of the United States Life Saving Service. Thence to one of the large dining halls where an elegant cold collation was served. The party then scattered over the ground- and buildings to enjoy the day as best suited individual inclinas tion. The main building, the Government building, the Art Gallery, the Horticultural Hall, the Mexican buildings, were all centers of attraction, the crescent badges of the S. D. A. being seen everywhere.

### FOURTH DAY.

Friday, April 3, was set apart for Clinics and the exhibition and explanation of Appliances. Mr. J. W. Selby, of the S. S. White Co., furnished the hall with all the necessary chairs, brackets, spittoons, etc. Simultaneous clinics drew together numerous animated groups.

Dr. Wm. N. Morrison illustrated the portion of the paper read by him, giving his method of filling root-canals with gold wire, through a very small opening, not more than one-sixteenth of an inch in diametor, in the centre of crown. The committee appointed to prepare the teeth selected those with the most crooked, curled and twisted roots that could be found in their collection, one in particular having a very remarkable double angle like a letter Z. The teeth were imbedded in blocks of plaster in such wise as to give no clue to the length, shape, or direction of the roots. The work was closely watched, and many were the predictions of the wire passing through the apical foramen on the one hand, or of failure to reach the apex on the other. The canals being first filled with gutta-percha, the slender gold wire was manipulated dexterously and rapidly, through an opening in the centre of the crown scarcely larger than a pin-hole. When the work was finished, the plaster was broken away, and the teeth eagerly broken open, when lo! not a single failure was to be recorded. In every case the gold was found to reach the apex exactly, to seal it securely, and with the gutta-percha forced into all the interstices the canal was perfectly filled All doubts were dispelled as to Dr. Morrison's ability to do all that he claimed. Whether anyone else could do it remained an open question.

Many gathered around the chair where Dr. E. Parmly Brown, of New York, with the electric mallet and spring pluggers of his own design, built up the crown of a left superior molar which had been nearly destroyed by malpractice with the file. The pluggers used were of a new design, the portion just above the point being tempered to a degree of elasticity that obviates all risk of crumbling down the margin of enamel. The separation between the teeth for knuckling was obtained entirely by the force of the gold driving the teeth apart at the point of contact. The contour of the crown, cusps, and masticating surface was perfectly restored.

Dr. Bonwill, of Philadelphia, gave a clinic with his mechanical engine and mallet.

Order was called at 3:30 P. M., that those having appliances on hand might have an opportunity of explaining them. Dr. H. J. McKellops, chairman of the committee, said that there were many interesting and valuable things on exhibition, that were worthy the attention of all present; that neither trouble, time, nor expense had been spared in getting them here; that some things were patented, some were "secret preparations," some were freely given for the benefit of all, but that he hoped respectful attention would be given to each. The articles on exhibition were: A novel and very simple articulator, invented by Dr. Westmoreland, of Columbus, Mississippi; a lathe head-piece (owner and inventor not present to explain); a tooth-powder bottle stopper, with a slot in the side for convenience of placing the powder on the brush, the invention of Dr. C. E. Kells, jr.; a regulating spring for expanding the arch, invented by Dr. E. S. Talbot, Chicago. The spring is of piano wire, the tension of the spring increased by being coiled in the middle, the spring interfering much less with articulation and mastication than either jack-screws or the coffin-plate. Dr. Miles, of Charleston, exhibited a new mouth-piece for facilitating the administration of nitrous oxide gas, and dispensing with the aid of an assistant; Dr. Williams, of Kentucky, a new motor-power for engines and lathes, which is perfectly noiseless, with band which cannot slip, that gives much greater power than any other, and capable of from ten to ten thousand revolutions a minute, as tested with the gauge; Dr. J. W. Smith, of Newport, Rhode Island, stiff paper disks with a rim of sand-paper for finishing cervical walls and proximal fillings; Dr. B. S. Byrnes, of Memphis, Tennessee, a new regulating appliance, or rather method, which commends itself by its extreme simplicity. It consists of merely a very thin, elastic band of 22 carat gold, hooked around the first molars, or most available posterior teeth, and bent into the interstices between the teeth. As the anterior teeth yield to the constant gentle pressure of the elastic band, and space is gained, the band is straightened and shortened as much as necessary and again indented to hold the teeth in the new position. This is repeated as often as necessary until the symmetry of the arch is attained. If a gap is to be closed a gold band is hooked around the teeth to be drawn together. If special action is desired upon any one tooth, a rubber block is placed under the gold band, but no rubber rings are used and no threads tied. Casts were exhibited showing the application of the principle and the very remarkable results obtained in a very short time with very little soreness of the teeth or discomfort of the patient, and very little time spent by the operator in applying the apparatus. The appliance was highly commended by Dr. McKellops.

Dr. J. A. Robinson, of Jackson, Michigan, exhibited rubber plates lined with fibrous metallic material, and also with vulcanizable gold, a material which does not corrode nor tarnish nor wear off. The injurious effects of contact of the rubber with the mucous membrane is thus avoided, while a beautiful finish is given to the plate.

Dr. Edwards, of Desmoines, Iowa, also exhibited specimens of black rubber plates made by a new method, which produces light, elastic plates, highly polished on both sides when they are taken from the flask, without the aid of any new apparatus.

Dr. J. R. Walker, of New Orleans, exhibited a modification of of the Coffin plate, having the spring so adapted as to be attached to the front teeth, to draw them in while expanding the arch laterally. An interesting sketch was given by Dr. McKellops of Dr. Coffin's experience in "giving away" a good thing. At the Medical Congress in London, in 1881, when he gave this appliance to the profession, he had on exhibition a peck measure full of plates that had done duty in more than twenty-five thousand cases. He had more than he could do with the help of two or three men, but after he had so generously demonstrated his method, everyone took it up, and his business fell away from him entirely when he no longer had the monopoly.

Dr. Bonwill exhibited his mechanical mallet and engine, explaining the difference between his and all others, the disadvantage of his electric engine and mallet being the necessity on the part of the operator for a thorough knowledge of chemistry and the laws governing electricity. The new engine and mallet also greatly expedites the work, enabling the operator to do in forty-five to seventy minutes what would otherwise require four or five hours.

Dr. Morrison exhibited and explained some very interesting specimens of Japanese dentistry; plates with cusped teeth, carved from hard grained wood, and stained to order, *black* for married women, as a mark of distinction, and all for two or three dollars a set. Also Japanese tooth brushes, of banyan root; the end beaten or bruised till the fibres take the semblance of a paint brush.

Dr. Morrison also exhibited a gold crown that had been presented to the Southern Dental Association fifteen years ago, but which had been in use three years, previous to that date, thus invalidating the several patents on the gold crowns now on the market.

Mr. J. W. Lambert, of the St. Louis Pharmaceutical Co., exhibited samples of Listerine and Menthated Camphor, "proprietary medicines," but not *secret preparations* as the formula is given on the label of each bottle. Listerine, though favorably known in the medical profession for some time, has but recently been offered to the dentist, as a scientific compound possessing valuable antiseptic and disinfectant properties, and specially adapted to dental and oral surgery on account of its absolute safety and freedom from objectionable odor and taste.

Mr. Wm. Evans, of McKesson and Robbins, New York, exhibited samples of the various preparations of cocaine, now being so largely experimented with as an obtundent of sensitive dentine, inflamed gum-tissues, exposed pulp, etc.

The Executive Committee announced the following applicants for membership:

T. J. Knapp, New Orleans.
Wm. Crenshaw, Vermillion, La.
R. E. Watkins, Eutaw, Ala.
A. E. Wofford, Starkville, Miss.
C. R. Rencher, Enterprise, Miss.

Who were duly elected, and their names placed on the roll.

Adjourned to meet in regular session 7:30 P. M.

Friday, April 3d, 7 P. M.—B. H. Catching, Third Vice-President, in the chair.

Report of the Committee on Dental Literature called for.

*Dr. B. H. Catching*, Atlanta, Chairman, read a valuable report, of which the following is a synopsis:

The literature of a profession gives it reputation and increases its usefulness. It is the only true index of merit; a guide to success and a terror to empiricism; revealing a past, improved in the present, to be perfected in the future--familiarity with it improving; ignorance degrading: a perpetual teacher, never graduating its pupils, but constantly adding to its course. He who lays aside his text-books, satisfied with his own acquirements, folds a bandage over his eyes that causes him to stumble, and binds himself with fetters that prevent all further progress. A few years back, illiteracy was the rule, for text-books were few and journals rare; but now, literature of the highest character is within the reach of all. Dentistry is not to be ashamed of its literature. It stands abreast with all the learned professions. One field of great importance is comparatively neglected: viz., materia-medica and therapeutics. The cabinet of the average dentist contains only creosote, carbolic acid, tannin, and arsenic—not entirely his fault; colleges teach students to display their handiwork, rather than instruct them in dental therapeutics. A new era is dawning--the dental student will soon be master of Garretson's system. One common degree will cover all specialties.

This is the electrical age, not only physically but mentally also. In the current news of the day, we know at the breakfast hour what transpired yesterday in the farthest corners of the globe. In the literature of science we are too slow for the age. We must sever the last link that binds us in the fetters of the past. Monthly journals are too slow; we must have a weekly dental journal. It must come from the profession, by the profession, for the profession.

*Dr. Brown*—As a guest of the Southern Dental Association, I recollect saying, on one occasion, that I was not quite full enough for utterance. Although I have been magnificently treated on the present occasion, I am not *too full* for expression. I have but a few moments to spare, however, so don't get discouraged. I shall not talk long. I am sorry to meet so few men from the North. I am sorry *for them*, for they do not know what they are losing. It has not been properly advertised. It would not be one hundred thousand men ; it would be an army of a million men that would come and capture New Orleans with the arms of friendship and love, if they knew the welcome they would meet, and what there is here for them to see. My train is probably whistling. I must bid you adieu. (Applause.)

The subject of Dental Literature resumed.

*Dr. E. S. Chisholm* (Tuscaloosa,) read a paper of which the following is a synopsis :

Dentistry is one of the learned professions and boasts its periodical literature. It is a bright mark of progress that our sayings and doings cannot be compiled into book-form and got out of the hands of the printer before "the latest" is already read in the journals and laid on the shelf. A recent Index of Cosmos gives a good idea of the variety of subjects treated. Every new invention, discovery, or idea reaches us promptly through our journals, whether the motive of the author be gain or fame or the love of his profession. "Supply and demand" control here as in all other business. Journalistic literature is an active potent agent in cementing into harmonious work the entire brotherhood ; in upbuilding a structure of scientific dignity. In our journals we are distinctive. In our text-books, much that we claim as ours, is borrowed from our neighbors, medicine and her specialties. Honor to whom honor is due.

Our fundamental knowledge of anatomy, physiology, pathology, we owe to medicine proper, though now common to both professions. We travel the same high road but switch off on our specialty, oral and prosthetic.

We are now far in advance of the medical profession in their application to the mouth. We borrow what she can give without loss to herself and return with usury. Many sciences also, as Chemistry, Botany, Metallurgy, have contributed to our literature. Their foundation principles were known before our profession had an existence, but we have enlarged and elaborated upon them. We now have twenty-one journals, exclusively dental, or from eight hundred to one thousand pages per month, or twelve thousand per year, exclusive of society transactions, essays, pamphlets, and advertising circulars. Our text-books are not deficient in quantity. Eighteen new volumes were added to the list during the past year; entirely new, or enlarged and clad in a new suit of the latest fashion.

Of these will only mention:

"Dental Vade Mecum," James Hardy.

"Student's Guide to Dental Anatomy and Surgery," Henry Sewell, London.

"Practical Treatise on Mechanical Dentistry," 3rd Edition, Joseph Richardson.

"Oral Deformities," Norman W. Kingsley.

"The Mouth and the Teeth," J. W. White.

"Letters from a Mother to a Mother," 2nd Edition, "Mrs. M. W. J." (Wife of a dentist.)

"Plastics and Plastic Fillings, 2nd Edition, J. Foster Flagg.

Harris' "Principles and Practice of Dentistry," 11th Edition.

"Dictionary of Medical and Dental Terminology," F. J. S. Gorgas.

"Operative Dentistry," 4th Edition, Prof. J. Taft.

Will not attempt comparison or contrast. Each and every one deserves a place in the library of every dentist.

War has been waged against the advertising pages of the journals. Those who complain the most of this are those who receive them free as an advertisement from the depot which issues them, and who otherwise would never see them. The clothes do not make the man; it is the character. This feature is far from objectionable. It keeps us informed of the latest material improvements and inventions--the benefit is mutual. We need, not more writing but better writing, but the greater the number of pens, the wider the range to select from; the broader the range of experimentalism, the greater the aggregate of experimental science. Much that we once thought very simple is now found to be unfathomable; as the cause of caries; the histological character of tissues, etc. But few write well. Practitioners of large experience and the best practical knowledge have no time, and perhaps no talent for the pen; others with good ideas connot express them clearly or concisely; some hesitate for fear of the waste basket; there are also others who would keep all they think they know for themselves. Much of which we complain is due to lack of preliminary education. The threshold needs to be more carefully guarded against the entrance of illiterate young men; ignorant young men, whose only ambition is to make a living without work, and who consider the ability to shoe a horse, to put a backspring in a knife, indicates capacity for the dental profession. It is difficult to get such raw material to stand a two year's pupilage—much less a college course. From such sources we cannot expect respectable practitioners; far less, good contributors to dental literature.

DISCUSSION.

*Dr. Spalding*—Every profession is undoubtedly aided by its literature. In our own profession, periodicals and standard works are sufficient in quantity but deficient in quality. Progress must be sought in that direction. Our text-books are deficient in certain branches. In materia medica and therapeutics almost *nil.* In other lines they have grown almost beyond our needs. Text-books are usually written by teachers. We have depended on medical men as teachers, and consequently as authors, in those branches. They know comparatively little of their application to dental therapeutics. Very much of what we know is not in the text-books; it has been learned from experience, and recorded in our journals. Medical men, as such, are not qualified to teach, or write books for, dental students. The remedy? We must bring forward men from our own ranks to teach in these departments, and to furnish the text-books we require. All the chairs in every faculty should, as a rule, be filled by dentists. It has been said that if physiology is a science, the man who can teach physiology can teach dental physiology. Practically this is far from being a fact. Unless a man expects to become an author, he will not be thorough. There are very few thorough physiologists, even among medical men. Education in the fundamental branches is only partial. Few are qualified to teach chemistry, as applicable to our profession. Unless a man is well grounded in first principles, he is not qualified to teach. He must also understand the practical application of the principles of science to our profession. We depend on medical men to teach our students what they themselves are ignorant of. We must draw from our own ranks to supply this deficiency.

*Dr. B. H. Catching*—In attending associations from year to year, I see exhibited gold, amalgam, mallets, engines, etc.; everything for manual use is brought before the body, but I have never seen a single volume of our literature laid upon our table; I hope our Southern Association will take steps to be furnished by publishers with copies of each and every new publication issued, to be examined by the profession at their annual meetings. I would have this made a part of the work of the Committee on Dental Literature.

*Prof. J. Taft*—Great improvement can yet be made, but when we look back to forty years ago, and note the improvement made in that time, in our literature, it seems almost a miracle. Then we had no literature. We had but three or four standard works, and the beginning of our periodicals; but now, see our monthly supply of dental literature! I think we may conclude that some progress has been made. Examine the pages of one of those early journals, and compare with one of to-day and again we see the progress.

Our literature needs improvement in its literary aspect. Our permanent work—our text-books—have not kept pace with our journalism; but new men are using strong pens, and our text-books will soon equal those of other professions. Every avocation has its special literature. We must train up writers for our profession. Every man should keep a record of every step in advance. Young men should be pressed to engage in this work. If all would do this, our literature would speedily improve. Many who now write are poor writers, it is true, but we should blame no one who does his best, but encourage him to further efforts. Reference was made in the paper to lack of suitable works in certain lines. Let those who think it ought to be better strive to make it better; there is no one to hinder; the way is open. Most of the books we have are designed for those already experienced; there are no elementary books; no clear simple books for beginners. That is the great need of to-day. It is an easy matter for those in practice to give information for those who are experienced, but it is not so easy to write for beginners. There is no elementary work on anatomy. If any one can be stimulated to prepare graded books for our profession, he will have attained a great desideratum. We need elementary works on anatomy and physiology. Would you put Gray, as the first book, in the hands of a young dental student? We should have an elementary work designed for our own students. The common-school-books of to-day are better than those used in our colleges. We should have a series of brief works that a student could readily master and retain. The nomenclature of anatomy and pathology ought to be improved. It should be anglicized. The student needs to be familiar with two or three extra languages. The bones and muscles should have plain English names. Students must have helps to more rapid progress. What if our periodicals do contain some advertising matter? That is no disadvantage. If a man pays for 40 pages of literary matter, and gets, for nothing, 50 pages more, which tell him where to get what he most needs, is he the loser? Some men turn and look for the new advertisements first thing, and then examine the literary portion if they have time left! Most of the journals are owned and published by the men who manufacture the goods you must have; they could not be sustained independently.

*Dr. J. R. Patrick*—I think Prof. Taft's position in regard to elementary works in our colleges, not tenable. There are elementary books in all our high schools, and a youth has no business in a college who has not already mastered these elementary works. Gray's Anatomy is plain and simple, and told in few words. Nothing could be plainer.

*Dr. J. R. Walker*—What is most needed is elementary instruction in hygiene in our primary schools. Such teachings as, when put into practice, will enable the scholars to enter the high-school, and go thence to our colleges, with sound minds in sound bodies. There is much lack of primary education in hygiene. Success in after-life depends on the health of the body as well as on the cultivation of the brain.

*Dr. O. Solomon* (New Orleans)—I am surprised to hear the depreciation of American literature. No nation on earth has what America has. German and French dental literature consists almost entirely of translations and extracts from American journals. When I came to America I thought I knew something about dentistry. I had studied in Paris, and in Berlin; but when I got to Baltimore I found I knew nothing. As to the advertising pages, if a man has profound knowledge, his dirty coat is a matter of little consideration. The German and French journals have no advertising pages, but neither have they any reading matter worth reading.

*Dr. E. S. Chisholm*—Our literature is just what we make it; it is the reflex of ourselves; it is evolved from the demand. We pay for it because we feel that we need it to help us in doing our best for our patients. It is a fact well-known to all who teach science that popular teachings are not correct. We must learn the first principles, in all branches, by the inductive system, gradually growing and broadening out.

*Prof. J. Taft*—The majority of our students, coming from the high-schools, where the elementary sciences are popularly supposed to be taught, have but the merest smattering: nothing that prepares them to take up Gray as a starting point. A student in mathematics is not given algebra or the third part arithmetic to begin with. He must start with the elementary principles; and so it is in all branches. As geographies and arithmetics are graded, so must the sciences have graded text-books: from the primary schools, up through the high-schools, to the college. Prof. Ford and others have prepared question-books, and the students study them out, taking the question-books as a guide to study, and the text-books as statements of facts. We should have little primers of 20 or 30 pages, of elementary principles, in which the student should be as thoroughly grounded as in the alphabet or multiplication table. He should have from ten to twenty preliminary lectures, before undertaking laboratory work. Though foreign journals may translate or copy our record of experiences, we are indebted to them for the fundamental principles of science underlying our practical work. Our foreign brother is warranted in speaking more freely on this point than I can do.

*Dr. Spalding*—I would call the attention of the Association to an illustration of the principle laid down. Gray's Anatomy is regarded as the best text-book on anatomy; the work of a most celebrated anatomical author. But turn to the pages on Dental Anatomy, and you will find a mere reproduction of that which written by Goodsir many years ago. This fossilized statement has been handed down without alteration or comment; there is only one chapter, and that one entirely erroneous from the modern standpoint. Must we wait for some medical man to study this subject out and write it up for us? No; we must draw upon our own resources, from our own ranks. That is the only way out.

*Dr. Patrick*—Gray's Anatomy is the text-book for the general practitioner of medicine; for the professional man. It does not purport to be a guide to any specialty, as of the eye or the ear or the throat. There are other text-books intended for men who are well-informed but not professional. Çarabelli (Vienne, 1826) gave us a valuable work in the beginning of the century, though no credit is given him. He took up the work where John Hunter left off. It is a work of world-wide celebrity, as authority for comparative anatomy. There is no lack of text-books for professional men.

*Dr. A. W. Harlan* (Chicago)—The members of this Association are not schoolboys to be taught the rudiments. The trouble is not in the lack of text-books; it is in the lack of study of those we have. The great trouble with text-books is that they are too truly elementary; they are not exhaustive. There is too great a multiplication of primers. Men think they know it all, and study no further. Science is not to be taught by primers—we have health primers, science primers, biological primers, etc. They contain a mere smattering, and put forth insignificant ideas. Revised editions of the books we have and the study of those books by the younger men is what is needed.

*Prof. Taft*—The class of books that Dr. Harlan names is not what I referred to. The primers serve a good purpose. His argument would cut down the progressive, inductive system. Gray's Anatomy was not written under the histological light of to-day. It contains much that requires modifying. I agree with Dr. Catching that copies of the new publications should be brought before the association. That in itself would be a good report on dental literature. It would make a more positive impression than a written report. Would like to see such a report at every meeting.

*Dr. B. H. Teague* (Aiken, S. C.)—I think it only right that this Association should take public recognition of the work of Mrs. M. W. J.; a work that has not only a national reputation, but a world-wide fame. I move that we tender the thanks of this Association to the author of the book entitled "Letters from a Mother to a Mother."

*Prof. J. Taft* moved an amendment adding that the Association recommend the book to all practicing dentists for circulation.

*Dr. C. W. Spalding*—Having had an opportunity of examining a copy of the recent revised edition, I would say that it does not do justice to the author. The workmanship does not correspond with the contents. The work itself is the very best of its class; has no equal, in my judgment, for placing in the hands of mothers.

The motion, as amended, passed unanimously, and was ordered so recorded.

*Prof. Taft* moved that the author be requested to prepare a revised edition free from the errors and faults referred to. Had examined the book and was prepared to endorse what Dr. Spalding said of its value. It was intended for distribution among patients, but every practitioner ought to read it. Moved that the author be requested to prepare an edition free from error.

*Dr. C. W. Spalding*—Though agreeing with the sentiment, would doubt the expediency of the measure, as an edition of 5,000 was just from the press, and the resolution would imply censure of the publishers, as the faults were faults of workmanship and typographical errors, etc.

"Mrs. M. W. J" was introduced to the members of the Association, and in a few words expressed her gratification at the reception her work had met and the kind words spoken, explaining that the text had been very carefully revised for the new edition, and that the typographical and other errors were entirely due to the haste in which the new edition had been issued in order to have it out in time for examination by the Association; that haste, however, having defeated its own object.

The motion of Dr. Taft was withdrawn.

The subject of Dental Literature was passed.

Dr. H. J. McKellops desired to call attention to the subject of the Code of Ethics—that he should probably tread on somebody's toes, but was only sorry for their corns! He wished to call attention to the matter of giving certificates—a subject to which he had been giving some attention. He knew that itinerants were in the habit of cutting out these certificates and pasting up whole columns to which they call the attention of patrons. Many of our own members have their names in print, attached to recommendations of articles of which it is very doubtful if they know the constituents. Look at the list of certificates to Holmes' Sure Cure! Do they know what this is? I propose to buy a bottle and present it to our distinguished chemist, Dr. Harlan, for analysis. I have fought this practice for years. If you want to get your names honorably before the public, buy a quantity of the books of the lady just mentioned, put your name on them, and distribute them far and wide. It will be better for yourselves, and good for your patients.

Report of the Committee on Clinics called for.

*Dr. McKellops* moved that the subject be passed as the time was very short, and there was much business that must be transacted—the election of officers; selection of time and place of meeting, etc.

Carried.

Proceeded to ballot for time and place of meeting.

Nashville was selected, the time being fixed for the fourth Tuesday in May, 1886.

Drs. E. Telle, New Orleans, and M. S. Read, Corsicana, Texas, were elected members and enrolled.

The election of officers was now had, resulting in the election of

Dr. C. W. Wardlaw, for President.

Dr. B. H. Catching, of Atlanta, Ga., First Vice-President.

Dr. J. Rollo Knapp, of New Orleans, La., Second Vice-President.

Dr. E. D. Hammer, of Galveston, Texas, Third Vice-President.

Dr. E. S. Chisholm, Tuscaloosa, Ala., Corresponding Secretary.

Dr. R. A. Holliday, of Atlanta, Ga., was re-elected Recording Secretary.

Dr. H. A. Lawrence, of Athens, Ga., was re-elected Treasurer.

Dr. W. H. Morgan, of Nashville, Tenn.; Dr. G. F. S. Wright, of Columbia, S. C., and Dr. W. H. Richards, of Knoxville, Tenn., were elected as the Executive Committee.

The newly elected officers were then inducted into office.

Dr. Jas. S. Knapp conducted the newly elected President to the chair. In a few well-chosen words, Dr. Wardlaw returned thanks for the honor conferred, begging the cordial support and co-operation of the members to make the next meeting equal the present in interest and benefit; proposing to make his administration a working one. He hoped that all members of committees would willingly fulfil their duty, or give early intimation of inability to do so—let us come up as a host supporting and helping each other, making this a great society for good.

"Mrs. M. W. J." was elected an Honorary member of the Association; the Secretary requested to give formal notification of the same.

The following resolutions were introduced by Dr. J. R. Walker, of New Orleans, and unanimously adopted:

*Resolved*, That the Southern Dental Association desires to extend a vote of thanks, in token of its profound appreciation of the very generous action of the Board of Management, the Trustees and Faculty of Tulane University, in affording to us and to the National Board of Dental Examiners the free use of their hall and committee rooms, day and night, during the full term of our meetings. Also to Mr. J. W. Selby, for his liberality in furnishing the hall with the chairs and other appliances necessary for clinics. Also for his generous assistance in receiving and entertaining the members of the Association. Also to the press of the city of New Orleans for their enterprising daily reports of the proceedings.

*Resolved,* That the thanks of the Association be tendered the retiring president, Dr. A. O. Rawls, for the able and judicious manner in which he has performed the onerous duties devolving upon him during his term of office.

Dr. Clifton, of Waco, Texas, moved that the thanks of the Association be tendered the dentists of New Orleans for the pleasant entertainment at the World's Exposition. Carried.

Dr. Walker moved that the Secretary and Treasurer be included. Carried.

Adjourned, to meet in Nashville, Tenn., on the 4th Tuesday in May, 1886.

